# Ovarian tissue bank for fertility preservation and anti-menopause hormone replacement

**DOI:** 10.3389/fendo.2022.950297

**Published:** 2022-09-09

**Authors:** Jing Chen, Yan Han, Wenjie Shi, Xiaohong Yan, Yingying Shi, Ye Yang, Hong Gao, Youzhu Li

**Affiliations:** ^1^ Department of Reproductive Medicine Center, The First Affiliated Hospital of Xiamen University, School of Medicine, Xiamen University, Xiamen, China; ^2^ The Assisted Reproduction Department, Yichun Maternal and Child Health Hospital, Yichun, China; ^3^ University Hospital for Gynecology, Pius-Hospital, University Medicine Oldenburg, Oldenburg, Germany; ^4^ Reproductive Medicine Center, Women and Children’s Hospital, Xiamen University, Xiamen, China; ^5^ Department of Clinical Medicine, School of Medicine, Xiamen University, Xiamen, China

**Keywords:** fertility preservation, ovarian tissue cryopreservation, transplantation, hormone replacement, anti-menopause

## Abstract

Traditional fertility preservation methods such as embryo or oocyte cryopreservation cannot meet the needs of a cancer patient or for personal reasons. The cryopreservation of ovarian tissue can be an alternative and has become a hot spot to preserve fertility or hormone replacement. The freezing of ovarian tissue can be carried out at any time without ovarian hyperstimulation to retrieve follicles. It is an ideal strategy to preserve reproductive function in children, adolescent cancer patients, and patients who are in urgent need of cancer treatment. With the increasing demands of women with premature ovarian failure or in menopause, ovarian tissue transplantation is also an alternative for hormone replacement that can provide physiological doses of hormone levels, which can avoid a series of risks such as thrombosis, breast cancer, or other hormone-dependent tumors, caused by oral hormone replacement. Hence, ovarian tissue banking can be regarded as a mainstream strategy for fertility preservation and anti-menopause hormone replacement in further clinical investigation.

## Introduction

Humans are living longer today, and at the same time, people have higher expectations for the quality of life. The duration of the fertility of females is short. When females reach 50 years of age, they will lose their fertility function. Ovarian tissue cryopreservation (OTC) has been encouraged by many scientific and medical communities to restore female reproductive function and recover endocrine function. The first indication of OTC is for the cancer patient to reestablish their fertility function, and the second indication is hormone replacement for menopausal patients. If a patient is diagnosed with malignant cancer, the ovarian tissue can be frozen before radiotherapy or chemotherapy. After the disease of the patient is cured, the follicles can be isolated, then embedded in an artificial ovary, and auto-transplanted back into the body to mimic the natural ovary to provide the patient with the hormone level to conceive their own genetic offspring and restore their physiological hormone state (1).

According to the statistics by the International Cancer Research Agency, the incidence rate of female cancer patients under 44 years old was up to 65%. Meanwhile, the survival rate has increased rapidly and nearly 70% have been cured of their original cancer due to the advancement of medical technology (2). But cancer therapy, including chemoradiotherapy and radiotherapy, may lead to irreversible ovarian failure and fertility loss that may decrease the quality of life (1). Scientists and clinicians advocate OTC before cancer therapy to preserve fertility and endocrine function in female cancer patients. Cheng et al. cryopreserved the ovarian tissue of a 26-year-old breast cancer woman before cancer therapy to preserve fertility. This patient is at a high risk of premature ovarian failure but also has a higher survival rate of recovering ovarian function (3). Ruan et al. also performed OTC in a patient with myelodysplastic syndrome (MDS) before chemotherapy, and these ovarian tissues were autografted back into the body after 2 years of disease cure. Three months later, the ovarian function was recovered and 1 year later, pregnancy occurred spontaneously (4).

OTC is an expectable method to recover ovarian endocrine function and to delay menopause. Nearly 90% of estradiol in the human body comes from a pre-ovulatory follicle, so the follicles are essential to maintaining endocrine function (5). Menopause accounts for nearly half of the life of women. Females in menopause not only lose fertility function but also endure menopausal symptoms on physical and psychological levels, such as hot flushes, neurasthenia, osteoporosis, and hypertension (6). Oral hormone replacement therapy (HRT) for the menopausal syndrome is currently commonly used in clinical practice. But it is difficult to determine the suitable administration dosage and dosing frequency of the drug compared with the hormone produced by the body itself. Additionally, there are many side effects of long-term oral HRT, such as the increasing incidence of hormone-dependent cancers, stroke, thromboembolism, and heart disease (7). Janse et al. have shown that transplantation of cryopreserved ovarian tissue in women with a low level of anti-Müllerian hormone can also maintain the graft function for about 5 years because when the ovarian reserve decreases, the rate of activation of primordial follicles in the ovary will slow down (8). Hence, OTC can be an alternative strategy for menopausal patients who may postpone menopause as well as hormone replacement therapy.

Since OTC can be used for recovering fertility, hormone replacement therapy, and postponing menopause. Many scientists have introduced OTC technology and resulted in more than 150 babies, with a 95% recovery rate of endocrine function, and 33% pregnancy rate (9), and the span of graft function can be maintained for 4–5 years, ovarian activity can be recovered within 4 months after transplantation (10). We review the literature which has been performed by different research groups about OTC, which illustrates and summarizes the protocols of OTC for a better transition from research to clinical application. OTC involves several sequential steps: ovarian tissue acquisition, cryopreservation, and transplantation.

## Ovarian tissue acquisition

The ovary is constituted by two main parts: the cortex in the outer and the medulla in the inner. The follicles, granulosa cells, and interstitial cells are laying in the cortex, while blood and lymphatic vessels lay in the medulla. Therefore, the ultimate goal of OTC is to preserve the primordial follicles, which are located in the cortex of the ovary (11). Usually it is recommended that to take one-fourth to one ovary or part of both ovaries by laparoscopy procedure, that depends on the ovarian function of the patient and the purpose of OTC. Ovarian tissue preparation also needs to remove the medulla part and cut the remaining ovarian cortex into pieces that allow cryoprotective agents (CPAs) to quickly penetrate into the tissue. Gavish et al. found that the best thickness of ovarian tissue is 1–2 mm. They compared the thickness of 1–2 mm *vs* 0.5–0.9 mm. Although thinner tissue is favorable for cryopreservation, thinner tissue can accelerate the activation and depletion of the follicle pool, thereby reducing the functional span of the transplanted tissue (12). The thickness of nearly 1–2 mm of ovarian cortex also allows cryoprotectants to penetrate, diffuse into follicles, and produce a higher cooling rate to minimize freezing and toxic damage. The area of tissue is dependent on the ovarian function. For young women or PCOS patients, the suitable tissue area is 3 × 3 mm, 5 × 5 m for moderate ovarian function, and 15 × 5 mm for poor ovarian function. Because the tissue is too small, more follicles are activated, and it is also difficult to fix and easy to move around during transplantation, which is not conducive to revascularization (13). By the way, the follicle in the ovarian cortex is mainly primordial follicle, but if there is a developed follicle in the cortex, we should aspirate it before freezing, because the follicular fluid in the developed follicle will format the ice crystal that may destroy the structure of ovarian tissue, and these aspirated developed follicles can also be cryopreserved for IVF (14).

## Technique for cryopreserving ovarian tissue

Cryopreservation of ovarian cortical tissue is a complex and challenging project because it contains many tightly-connected groups of cells, like follicles with oocytes, which are surrounded by granulosa and theca cells, stromal cells, and blood vessels. Hence, it is vital to choose an optimal cryopreservation technique for OTC, which includes several steps below for short: Firstly, expose tissues in CPA to remove intracellular moisture and prevent ice crystal formation from damaging cellular structures. Then cool the tissue to a sub-zero temperature and store it for a long time. After thawing, dilute and remove cryoprotectant to return to the physiological environment for further growth. Currently, there are two common techniques for human ovarian tissue cryopreservation: slow freezing and vitrification (**Table 1**). There are at least two main kinds of side effects that occur during cryopreservation. The first side effect of slow freezing is ice crystal formation from intracellular moisture. The formation of ice crystals may lead to a high concentration of cytoplasm that causes damage to the cells. The second side effect is the toxic effect of the cryoprotectants. When the cooling rate is too slow, causing the cells to be exposed to the cryoprotectant for too long a time, or the concentration of the cryoprotectant is too high, which will have a toxic effect on the cells (22).

**Table 1:** Clinical practice of ovarian tissue cryopreservation.

Patient	Age(Years)	Tissue dimension (mm)	Cryopreservation method	CPA	Carrier into liquid nitrogen	Transplantation	Outcomes	Ref
ovarian abscesses N = 1	18	7–8 × 4 × 1-2	Slow freezing	DMSO	Cryogenic vials	Peritoneal pocket in the broad ligament	Follicle growth 24 weeks post-transplantation. One live birth	(15)
stage IV Hodgkin's lymphoma N = 1	25	12 × 4	Slow freezing	DMSO	Cryogenic vials	Peritoneal window beneath the ovarian hilus	Ovarian endocrine function restoration 5 months post-transplantation. One livebirth	(16)
benign ovarian surgery N = 10	16–34	4 × 4 × 1	Slow freezing	DMSO	Cryogenic vials	–	Decreased telomere length and increased senescence markers in ovarian tissue	(17)
malignant breast neoplasm N = 1	28	1–2 × 3–5 × 1–2	Slow freezing	PrOH Sucrose	Cryovial	Lateral pelvic wall	ovarian endocrine function restoration 6 months post-transplantation. One live birth	(18)
Malignant cancer N = 4	29–37	5 × 5 × 1	Slow freezing	DMSO Sucrose	Cryogenic vials	Between rectus muscle and sheath	Endocrine function was recovered 12–20 weeks post-transplantation and last for 3 months to 7 years. Four embryos for IVF	(19)
POI N = 37	28–48	5–10 × 5–10 × 1–2	Vitrification + IVA	EG Sucrose	Stainless needles of the Cryosupport	Underside serosa in one or both Fallopian tubes	Nine patients have follicle growth. Three pregnancies. Two live births	(20)
cancer survivors, POF patients N = 4	18–30	10 × 10 × 1–1.5	Vitrification	EG DMSO Sucrose	Thin metal strip	Denuded ovarian medulla	The longest functioning graft was 62 months and is still functioning. Two live births	(21)
cancer survivors, POF patient N = 9	18–30	10 × 10 × 1–1.5	Slow freeze	EG DMSO Sucrose	Cryovials	Denuded ovarian medulla	The longest functioning graft was 56 months and is still functioning. Nine live births	

## Slow freezing versus vitrification

**Figure 1:**
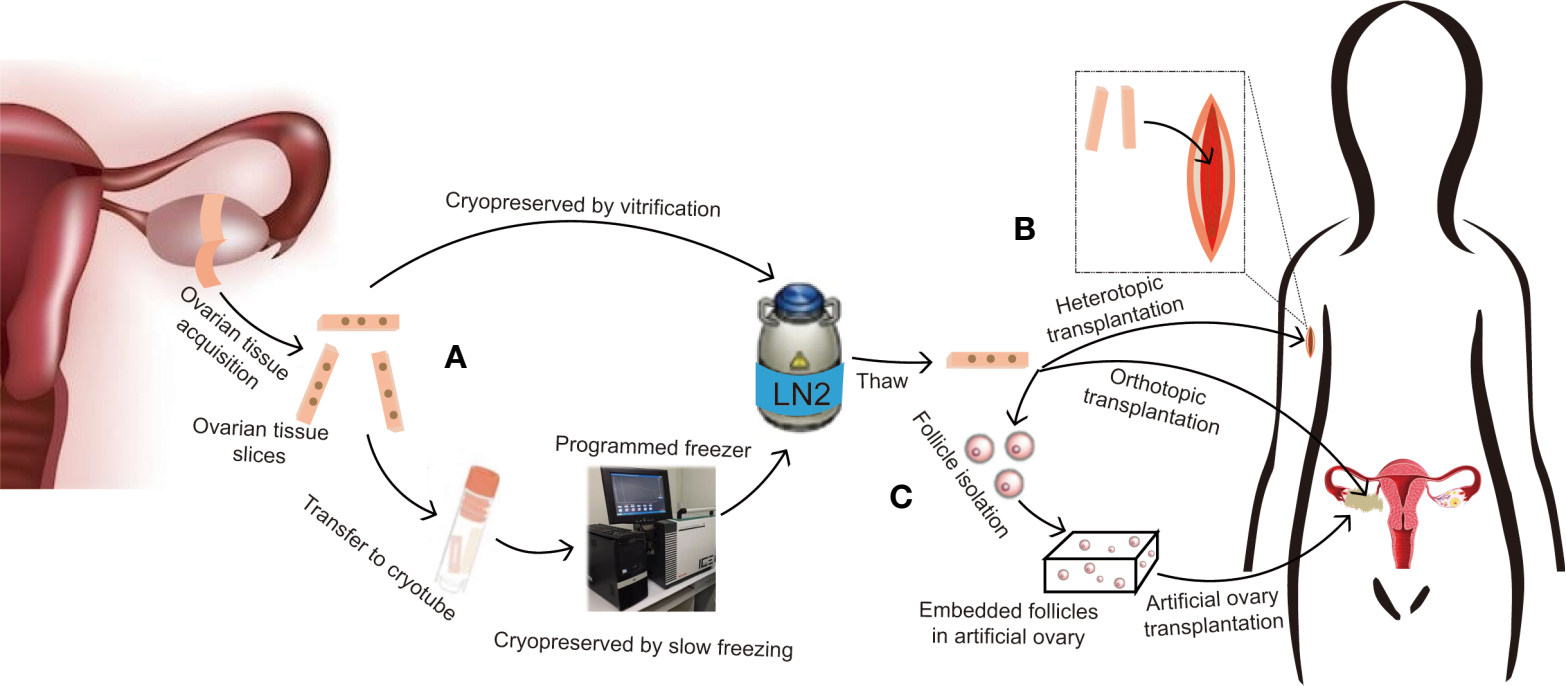
Protocol for ovarian tissue cryopreservation to preserve fertility and restore endocrine function. **(A)** If the patient is prepubertal or requires immediate chemotherapy with a potential risk of transmitting malignant cells, ovarian tissue slices are removed and long-term cryopreserved in liquid nitrogen by slow freezing or vitrification. **(B)** After thawing, if there is no risk of transmitting malignant cells, the ovarian tissue can be used for orthotopic transplantation for natural pregnancy and to restore endocrine function, or heterotopic transplantation for further *in vitro* fertilization or hormone replacement therapy only. **(C)** If there is a risk of transmitting malignant cells, follicles can be isolated from cryopreserved tissue and embedded in an artificial ovary, then transplanted into orthotopic or heterotopic sites for fertility preservation and endocrine function restoration.

Slow freezing, also known as programmed freezing, is commonly used for OTC. This process requires dehydrating the tissue in a low concentration of CPA solution, then transferring it to a cryovial, which contains a freezing medium, and putting it into a programmed freezer that can be programmed to cool down by steps according to the predefined temperature drop, and finally storing it in liquid nitrogen (**Figure 1**). After warming, put the tissue into a gradually decreasing concentration of CPA to gradually rehydrate and remove the cryoprotectant from the tissues. Controlling the cooling rate and adding CPAs can protect cells from damage by balancing the osmotic pressure inside and outside the cells (2). Donnez et al. did slow freezing ovarian cortex of a woman with ovarian abscesses at 18 years old and auto-transplantation at 28 years old. Twenty weeks after transplantation, the ovarian function began to recover, and a healthy birth was delivered by IVF (15). Other researchers also give live birth to cancer patients with stage IV Hodgkin’s lymphoma and malignant breast neoplasm by slow freezing the ovarian cortex (16, 18). Kim et al. cryopreserved ovarian tissue using slow freezing from ten women with benign ovarian surgery. The morphology of follicles was similar between the cryopreservation group and the fresh group, but the number of primordial follicles was decreased after cryopreservation, and they also found some DNA changes in ovarian tissue after cryopreservation, such as decreased telomere length and increased senescence markers (p53, p16, and p21 proteins) (17).

Although slow freezing is currently a mature technology, the refrigeration equipment required for slow freezing is expensive, and the cooling speed is slow and time-consuming. Vitrification, on the other hand, with a supercooled rate that can directly glass-like solidify without ice crystal formation, needs a high concentration of CPAs, which is toxic to the tissue. For these reasons, the ultra-fast cooling rate makes the freezing process difficult to operate, which requires very high operator proficiency. These reasons limit the widely used vitrification method (23). The speed of cooling, the volume, and the viscosity of the tissue are the three leading factors that affect vitrification. Xiao et al. vitrified ovarian tissue with three different systems: an open system with needle immersed vitrification (NIV), a closed plastic system, and a closed silver system. The silver closed system can better vitrify follicles with higher vitality, better morphology, and less risk of infection (24). Suzuki et al. vitrified ovarian tissue combined with *in vitro* activation (IVA) for women with primary ovarian insufficiency (POI) and generated three pregnancies with two live births (20). Silber et al. compared slow freezing with vitrification for cryopreserving ovarian tissue and found that tissue by vitrification had no oocyte loss, whereas slow freezing resulted in nearly 50% loss. The morphological were similar, but the vitrification group has less DNA damage. Both groups have normal and healthy offspring (21). Isachenko et al. found the hormonal activity and follicle quality were similar in ovarian tissue cryopreservation by slow freezing and vitrification, but the GAPDH gene expression in the vitrification group was significantly decreased compared to slow freezing (25). Sugishita et al. compared slow freezing and vitrification methods by cryofreezing 50 ovarian cortical pieces and found the declined primordial follicle densities were similar in both groups, and the number of DNA damage, apoptosis, and intact primordial follicles were also similar in both groups (26). Galbinski et al. also compared cryopreservation of ovarian tissue by slow freezing or vitrifying in a metal closed system, and found that the number of follicles in both groups was lower than that in the fresh group, but the intact follicles in the vitrification group were higher, and the heat shock protein 70 kDa response—HSR in the slow freezing group was higher. Hence, they believed that both methods could be used for cryopreservation of ovarian tissue (27).

## Ovarian tissue transplantation

Ovarian tissue transplantation is classified as orthotopic and heterotopic transplantation according to whether there is a need for natural conception. Orthotopic transplantation means ovarian tissue being retransplanted in the pelvic cavity, such as the ovarian medulla, peritoneal window, and serosa of fallopian tubes. Heterotopic transplantation means ovarian tissue being replanted outside the pelvic cavity into places like the forearm, breast tissue, rectus muscle, abdomen, and subperitoneal tissue (19).

Orthotopic transplantation is commonly used in clinical practice due to its similar physiological environment as to the original, and it can also provide patients with a chance to conceive naturally. The recovery function of ovarian tissue after orthotopic transplantation is determined by the reconstruction of the blood supply. The ovarian cortex was in a hypoxic state during the first 5 days of transplantation, after which the hypoxic state gradually improved with revascularization. Revascularization depends on the blood supply of the transplantation site and the angiogenesis of the transplantation tissue (28). Establishing a transplantation window and inserting an ovarian tissue into the transplantation window before cryopreserved ovarian tissue transplantation is a key step to promoting angiogenesis (28). Another critical step is finding a vascularized transplant site. Ovarian tissue can be transplanted into ovarian incisions (subcortical or denuded medulla), peritoneal pockets, and subcutaneously in the abdomen, which have all been shown to be well vascularized (29). Finally, ovarian tissue can be fixed by interceed, stitches, and fibrin glue (30). Peritoneal pockets are ideal because of their vascularity and ease of manipulation. Before transplantation, it is best to check the patency of the fallopian tubes, and ovarian fragments should be transplanted on the side of the fallopian tube that is unobstructed (30). Donnez et al. replanted an ovarian fragment in a peritoneal window in the broad ligament near the ascending uterine artery and finally covered it with interceed. Growing follicles can be detected 24 weeks post-transplantation. Five oocytes were retrieved and one healthy male infant was born by IVF (15). Further experiments transplanted frozen-thawed ovarian fragments onto the ovarian medulla decorticated area. Nine months after grafting, the patient was tested pregnant and finally gave birth to a healthy boy (29).

Heterotopic transplantation may not be an ideal environment for follicle development due to differences in temperature, pressure, paracrine factors, and blood supply, but it also has some advantages. First of all, there is no need to undergo an abdominal operation, which can reduce the pain of the patient and the cost of the operation. Secondly, it can allow repeated oocyte retrieval at intervals due to its being less invasive and easy to monitor. Finally, it is an alternative to severe intra-abdominal adhesions. Kim et al. applied slow freezing over the ovarian tissue of women with malignant cancer for 1–10 years. After cancer therapy, they transplanted tissue through the skin incision and located between the rectus muscle and sheath. The endocrine function was recovered 12–20 weeks after transplantation and can last for 3 months to 7 years, and even resulted in four embryos by IVF (19). Stern et al. performed slow freezing on the ovarian tissue of a 17-year-old female with non-Hodgkin’s lymphoma (NHL) and transplanted it back to the pelvic sidewall and anterior abdominal wall subperitoneal after thawing. Ovarian tissue in both abdominal and pelvic sites can restore endocrine function and can also recover oocyte successfully (31). Another study transplanted frozen-thawed ovarian tissue into the forearm and abdominal wall. These two sites can also monitor the recovery of ovarian hormone secretion (32).

## Additives to improve transplantation survival

When frozen-thawed ovarian tissue is transplanted back into the body, it will first go through a hypoxia stage of about 5 days, and the hypoxia state will gradually improve after 5 days with the reconstruction of ovarian angiogenesis. Ovarian angiogenesis is complexly regulated by multiple vasoactive and angiogenic factors, of which hypoxia-related responses can promote angiogenesis by upregulating several growth factors. Among them, VEGF is a powerful angiogenesis promoter, and its expression can be upregulated 40–60 times in the transplanted ovarian tissue (33). Ten days after ovarian tissue transplantation, although the neovascular system has been rebuilt in time, reactive oxygen species (ROS) will be generated due to the tissue ischemia-reperfusion. The increase in ROS may induce protein, lipid, and DNA modification, which will lead to further cell damage (34).

Israely et al. transplanted ovarian tissue into prepared angiogenic granulation tissue, which was produced by wound healing. This is because granulation tissue can initiate the endogenous process of angiogenesis. The ischemic span was reduced by 24 h, which enables ovarian angiogenesis to be detected within 2 days after transplantation and significantly increases the number of healthy primordial follicles and the angiogenesis area of the graft (35). Sphingosine 1-phosphate (S1P) can activate the S1PR1-3 receptor of endothelial cells and regulate vascular development, antioxidants, and angiogenesis (36). Soleimani et al. regrafted the ovarian fragment together with S1P, and angiogenesis and follicle proliferation were dramatically increased, and less apoptotic. If S1P was combined with VEGF in the graft, twice as many follicles and angiogenesis were generated after transplantation (37). Another study also added bFGF to the graft and transplantation *in vivo*, higher follicle proliferation, increased angiogenesis, and less apoptosis were detected in the graft (38).

Mesenchymal stem cells (MSCs) can differentiate into endothelial cells and pericytes, which provide the necessary cellular components to stabilize newly formed blood vessels. MSCs can also promote angiogenesis by secreting growth factors (such as VEGF, bFGF, TGFβ, etc.) during hypoxia (39). Nearly 109 cytokines supported by MSCs can promote follicle recovery. Adipose tissue-derived stem cells (ASCs) are one type of MSC that can be easily harvested in large quantities through minimally invasive procedures such as liposuction. Moreover, ASCs retain their phenotypic and functional characteristics after long-term cryopreservation, which makes it possible to use autologous ASCs in the clinic. In a study in which Manavella et al. infiltrated the grafts with ASCs and transplanted them into the body, high concentrations of ASCs were found to significantly increase the vascular area after 14 days of transplantation (39). Yang et al. also added umbilical cord-derived stem cells (UC-MSCs) together with graft and then transplanted them into POF mice. Follicle recovery rate and angiogenesis were increased after 14 days of transplantation (40).

## Conclusions

Ovarian tissue cryopreservation and transplantation is now a highly demanded technology for fertility preservation and recover ovarian endocrine function, and can also allow the conceiving their own genetic offspring. Although ovarian tissue transplantation has been practiced by many experiments and has been achieved in more than 130 births, it still needs more technical and protocol improvements to find a suitable transplantation site, improve survival rate and outcomes, which can be practiced for further clinical treatment.

## Author contributions

JC wrote the manuscript and figures. WS and YH edited the grammar and revised the manuscript. XY edited the grammar and revised the manuscript. YS and YY reviewed the literature of manuscript. YL and HG conceived the framework of this review article, provided insights into the manuscript. All authors contributed to the article and approved the submitted version.

## Funding

This work was supported by the General Project of the Fujian Natural Science Foundation (No.2019J01565).

## Conflict of interest

The authors declare that the research was conducted in the absence of any commercial or financial relationships that could be construed as a potential conflict of interest.

## Publisher’s note

All claims expressed in this article are solely those of the authors and do not necessarily represent those of their affiliated organizations, or those of the publisher, the editors and the reviewers. Any product that may be evaluated in this article, or claim that may be made by its manufacturer, is not guaranteed or endorsed by the publisher.

## Abbreviations

OTC, ovarian tissue cryopreservation; MDS, myelodysplastic syndrome; HRT, hormone replacement therapy; CPA, cryoprotective agent; NIV, needle immersed vitrification; IVA, *in vitro* activation; NHL, non-Hodgkin’s lymphoma; ROS, reactive oxygen species; S1P, sphingosine-1-phosphate; MSCs, mesenchymal stem cells; ASCs, adipose tissue-derived stem cells; DMSO, dimethyl sulfoxide; PrOH, 1,2-propanediol; EG, ethylene glycol; POI, primary ovarian insufficiency; POF, premature ovarian failure.
